# Childhood conduct problems, potential snares in adolescence, and problematic substance use in Brazil

**DOI:** 10.1111/jora.70099

**Published:** 2025-11-19

**Authors:** Fauve Stocker, Jon Heron, Matthew Hickman, Fernando C. Wehrmeister, Helen Gonçalves, Ana Maria B. Menezes, Joseph Murray, Gemma Hammerton

**Affiliations:** ^1^ Population Health Sciences, Bristol Medical School University of Bristol Bristol UK; ^2^ Medical Research Council Integrative Epidemiology Unit at the University of Bristol, Population Health Sciences, Bristol Medical School University of Bristol Bristol UK; ^3^ Health Protection Research Unit in Behavioural Science and Evaluation (HPRU BSE) at the University of Bristol, Population Health Sciences, Bristol Medical School Bristol UK; ^4^ Postgraduate Program in Epidemiology Universidade Federal de Pelotas Pelotas Brazil; ^5^ Human Development and Violence Research Centre (DOVE), Universidade Federal de Pelotas Pelotas Brazil

**Keywords:** 1993 Pelotas Birth Cohort, Brazil, conduct problems, counterfactual mediation, substance use

## Abstract

Childhood conduct problems are associated with problematic substance use in adulthood; however, little is known about what might explain these associations outside of high‐income countries where the majority of research is conducted. Data were analyzed from 4599 young people from the 1993 Pelotas Birth Cohort in Brazil. The exposure was conduct problems (age 11 years). Outcomes included hazardous alcohol consumption and illicit drug use (age 22 years). Mediators included police arrest (by age 18 years), gang membership (ages 18 and 22 years), and school noncompletion (by age 22 years). We performed counterfactual mediation using the parametric g‐computation formula to estimate the indirect effect via all three mediators simultaneously. After adjusting for confounders (including hyperactivity problems), conduct problems were weakly associated with police arrest (OR [95% CI] = 1.45 [0.97, 2.16]) and school noncompletion (OR [95% CI] = 1.46 [1.22, 1.74]), but not with gang membership. Police arrest and gang membership were associated with illicit drug use (OR [95% CI] = 3.84 [2.46, 5.99]; OR [95% CI] = 7.78 [4.30, 14.10], respectively) and with hazardous alcohol use (OR [95% CI] = 1.60 [1.08, 2.38]; OR [95% CI] = 1.88 [1.07, 3.30]), after adjusting for confounders (including hyperactivity and emotional problems). There was no association between school noncompletion and either outcome after confounder adjustment. There was little evidence for an indirect effect of conduct problems on hazardous alcohol use and illicit drug use via all three mediators after confounder adjustment. Findings highlight the importance of school professionals being aware of the risk for school noncompletion for those with conduct problems. Additionally, programmes designed to reduce substance use in Brazil should focus on young people involved in gangs, and in the criminal justice system.

## INTRODUCTION

Conduct problems refer to behaviors related to conduct disorder (norm‐breaking behaviors and violations of the rights of others) and behaviors related to oppositional defiant disorder (noncompliant, angry, and defiant behaviors). In Brazil, conduct problems are almost twice as prevalent as in high‐income countries (HIC), with an average prevalence of 21% compared to 13% in the U.K. and 11% in the U.S. (Murray, Anselmi, et al., 2013), with a higher prevalence even after accounting for measurement differences (Hammerton et al., 2019). There is robust evidence that childhood conduct problems are associated with alcohol and drug misuse in adulthood (Bevilacqua et al., 2018). Although the majority of evidence comes from HIC, a previous study comparing a British and Brazilian birth cohort found that associations for childhood conduct problems with hazardous alcohol use and illicit drug use in early adulthood were stronger in Brazil compared to the U.K. (Hammerton et al., 2019).

Problematic substance use places a significant burden on society as it is linked with alcohol dependency, road traffic accidents, injury, violence, and crime (Rehm et al., 2017). To reduce problematic substance use, it is important to identify risk factors, particularly those which might be causally related and also modifiable. An influential developmental taxonomy published by Moffitt in 1993 introduced the concept of snares that can trap adolescents into long‐term problematic behaviors beyond an age when most otherwise desist from these behaviors (Moffitt, 1993). Previous literature has suggested that interrupted education, gang membership and contact with the criminal justice system can act as snares trapping some adolescents into persistent problem behavior, including problematic substance use, when desistance is normative (Bevilacqua et al., 2018; Lahey, 2015; McGee et al., 2015; Widdowson et al., 2021).

Very little research has examined the importance of potential snares in low‐ and middle‐income countries (LMIC). This is particularly important to address in countries, such as Brazil, with a higher prevalence of both conduct problems (Murray, Anselmi, et al., 2013) and potential snares such as gang membership and school noncompletion (Higginson et al., 2018; UNICEF Brasil, 2021), compared to HIC. In LMIC settings, the processes for the association between conduct problems and later problematic substance use might differ substantially from HIC due to fewer supportive systems and different social contexts for children with conduct problems (Murray, Anselmi, et al., 2013; Murray, Cerqueira, et al., 2013). School noncompletion, gang membership, and contact with the criminal justice system were hypothesized to be particularly important in Brazil, compared to HIC, given their higher prevalence. These snares might be important targets to prevent young people with conduct problems from experiencing persistent problems into adulthood, given that they can harm health, undermine a successful transition into adulthood and also lead to social disadvantage (Moffitt, 1993).

Considering school noncompletion as a possible “snare” linking conduct problems and later substance use, in Brazil, repetitive grade retention is a very common practice (UNESCO, 2010) and is a strong predictor of dropping out of school (Rumberger, 1995). Longitudinal data show Brazilian children repeating a school grade by age 11 had increased odds of elevated conduct problems into mid‐adolescence (Martins‐Silva et al., 2022), and those with an externalizing disorder in adolescence had higher odds of grade repetition 3 years later (Hoffmann et al., 2021). The association between childhood conduct problems and school noncompletion is also well documented in HIC (Colman et al., 2009; Erskine et al., 2016; Gubbels et al., 2019; Kulkarni et al., 2021; Lau et al., 2023). However, it is not clear if there is a causal relationship between conduct problems and school noncompletion, with some studies suggesting it is confounded by inattention (Kulkarni et al., 2021). There is evidence that school noncompletion is associated with problematic behaviors in early adulthood, such as substance use (Crosnoe, 2006; Griffin & Botvin, 2010), and completing school by age 22 years was associated with lower odds of violent and nonviolent crime, even after accounting for school grade repetitions in Brazil (Martins et al., 2022).

Considering another possible “snare” following child conduct problems, youth gangs are more prevalent in Brazil than in most HIC and may offer a form of social capital and protection to adolescents who have failed to comply with societal norms (Higginson et al., 2018; Howell & Egley, 2005). Excessive alcohol, drug use, and interpersonal violence are common among gang members and becoming a member increases the chances of early initiation and frequent consumption of alcohol and illicit drugs (Swahn et al., 2010). There is evidence that childhood conduct problems are a risk factor for gang membership in both HIC and LMIC (Higginson et al., 2018; Howell & Egley, 2005; Peterson & Morgan, 2014).

Finally, contact with the criminal justice system can be a consequence of childhood conduct problems, given the strong links between childhood conduct problems and later crime in LMIC (Hammerton et al., 2019; Murray & Atilola, 2020). According to labeling theory, the stigmatizing effects of obtaining a criminal record are internalized by young people, resulting in self‐stigma, low self‐efficacy and self‐esteem, increasing susceptibility to long‐term problematic alcohol and drug use (Newman & Crowell, 2023).

Therefore, the potential snares of school noncompletion, gang membership, and contact with the criminal justice system were hypothesized to be important mediators given evidence that they can be consequences of conduct problems using data from both HIC (Colman et al., 2009; Erskine et al., 2016; Gubbels et al., 2019; Howell & Egley, 2005; Kulkarni et al., 2021; Lau et al., 2023; Peterson & Morgan, 2014) and Brazil (Hammerton et al., 2019; Higginson et al., 2018; Hoffmann et al., 2021; Martins‐Silva et al., 2022; Murray & Atilola, 2020) and are also associated with negative outcomes, including substance use (Crosnoe, 2006; Griffin & Botvin, 2010; Martins et al., 2022; Newman & Crowell, 2023; Swahn et al., 2010).

One of the challenges for understanding relationships between child conduct problems, potential snares, and later substance use is the possibility of bidirectional relationships and shared or distinct sets of confounders for each relationship. Addressing these challenges requires data from a study where young people are followed across the life‐course, with potential confounders assessed across multiple domains. In the current study, we examine whether school noncompletion, police arrest, and gang membership mediate the association between childhood conduct problems and later substance use using a population‐based, prospective birth cohort in Brazil: the 1993 Pelotas Birth Cohort.

We hypothesize (i) associations between conduct problems at age 11 years with police arrest at age 18 years, gang membership at age 18 and 22 years, and school noncompletion by age 22 years; (ii) associations between police arrest at age 18 years, gang membership at age 18 and 22 years, and school noncompletion by age 22 years with hazardous alcohol use and illicit drug use at age 22 years; and (iii) an indirect effect of conduct problems at age 11 years on hazardous alcohol use and illicit drug use at age 22 years via school police arrest, gang membership, and school noncompletion. We expect that potential confounders will weaken these indirect effects, particularly when accounting for childhood hyperactivity (Fergusson et al., 2007; Hammerton et al., 2019; Kulkarni et al., 2021; Luderer et al., 2021). Other hypothesized baseline confounders include sex, sociodemographic and health risk factors for conduct problems, parental depression and substance use, the quality of the parent–child relationship, parental separation, and neighborhood safety (Fergusson et al., 2008; Griffin & Botvin, 2010; Gubbels et al., 2019; Higginson et al., 2018; Howell & Egley, 2005; Locatelli et al., 2012; Mahedy et al., 2018; Martins‐Silva et al., 2022; Murray et al., 2010, 2015, 2018; Peterson & Morgan, 2014; Tavares et al., 2004). Hypothesized intermediate confounders include peer deviance and drug use and early substance use (Erskine et al., 2016; Fergusson et al., 2008; Gubbels et al., 2019; Heron et al., 2013; Higginson et al., 2018; Hingson et al., 2006; Howell & Egley, 2005; Mahedy et al., 2018; Moffitt, 1993; Peterson & Morgan, 2014; Pitkänen et al., 2005).

## MATERIALS AND METHODS

### Sample

The 1993 Pelotas Birth Cohort Study is an ongoing population‐based study designed to investigate the effects of a wide range of influences on health and development. Pelotas is a city located in the extreme south of Brazil, with an estimated population of 345,000 inhabitants, 93% of whom live in the urban area. All births occurring in the five maternity clinics in the town were monitored in 1993 (99% of births in Pelotas occurred in hospital). For the 5265 children born alive, only 16 mothers could not be interviewed or refused to participate in the study. The 5249 newborns, whose mothers lived in the urban area, were included in the cohort (81 were either twins or triplets; 50% were female, 50% were male, 77% had White mothers, and 23% had Black or mixed race mothers). The detailed methodology of this study can be found elsewhere (Gonçalves et al., 2018; Victora et al., 2008). During the perinatal study, mothers were interviewed to collect demographic, health and socioeconomic information about the family. Follow‐up visits were conducted in 2004–2005 (age 11; *N* = 4452 mothers; retention rate of 87.5%, after accounting for *N* = 141 deaths), 2008 (age 15; 4349 mothers; retention rate of 85.7%, after accounting for *N* = 147 deaths), 2011–2012 (age 18; 4106 mothers; retention rate of 81.4%, after accounting for *N* = 164 deaths), and 2015–2016 (age 22; *N* = 3810 young people; retention rate of 76.3%, after accounting for *N* = 193 deaths) (Gonçalves et al., 2014, 2018). Study data at age 22 years were collected and managed using REDCap electronic data capture tools (Harris et al., 2009). The perinatal study and each follow‐up were approved by the Research Ethics Committee of the Federal University of Pelotas School of Medicine. After being informed of the details of the study, participants signed a term of informed consent.

### Measures

#### Exposure: Conduct problems at age 11 years

Conduct problems were assessed at age 11 during an interview with the primary caregiver (usually mothers) using the Strengths and Difficulties Questionnaire (SDQ) (Goodman, 1997). The SDQ is a screening questionnaire for children's mental health problems that occurred in the previous 6 months and has been validated in Brazil (Anselmi et al., 2010; Fleitlich‐Bilyk & Goodman, 2004) using independently diagnosed psychiatric disorders. Five items form the conduct problems subscale, including “often has temper tantrums or hot tempers,” “often fights with other children or bullies them,” “often lies or cheats,” “steals from home, school or elsewhere,” and “generally obedient, usually does what adults request.” Responses for each item are “Not true,” “Somewhat true,” and “Certainly true.” The item “generally obedient” was reverse coded. All items were summed together (range 0–10) and the scale was dichotomized to create a binary exposure of low conduct problems (less than four) versus high conduct problems (four or more) according to the cut point on the newer 4‐band categorization (https://www.sdqinfo.org/py/sdqinfo/c0.py). We dichotomized conduct problems given our hypothesis that it would be high levels of conduct problems that might lead to extreme consequences such as gang involvement, school dropout, and police involvement. Thirty‐one percent of children at age 11 years had high conduct problems.

#### Outcomes: Hazardous alcohol use and illicit drug use at age 22 years

The 10‐item self‐report Alcohol Use Disorder Identification Test (AUDIT; Babor et al., 2001) was used to assess hazardous alcohol consumption in the past year at age 22 years. The AUDIT is a brief screening tool aiming to detect risky drinking and risk for alcohol dependence with high validity and reliability (Allen et al., 1997) and has been validated using diagnostic interview, physical examinations, and laboratory testing (Bohn et al., 1995). The AUDIT was dichotomized at a cut point of eight and treated as a binary variable in main analyses. The cut point of eight or more represents hazardous levels of drinking and has been validated in Brazil (Lima et al., 2005) using psychiatric diagnoses of alcohol use disorders. We dichotomized the AUDIT to provide a measure that represents problematic levels of drinking, given that low levels of alcohol use are normative in young adulthood, and our hypothesis was about the presence of snares that can trap young people into persistent problematic behaviors. Twenty‐two percent of young people reported hazardous alcohol consumption at age 22 years. In a sensitivity analysis, two AUDIT subscales were used as dimensional measures of alcohol consumption (sum of first three items on consumption; range 0–12) and alcohol problems (sum of remaining seven items on negative consequences experienced when drinking alcohol; range 0–28).

Illicit drug use was assessed using the same self‐report questionnaire at age 22 years which included questions about lifetime use of cannabis, cocaine, crack, amphetamine‐type stimulants, nitrous oxide or other inhalants, hallucinogens, opioids, and other injected illegal drugs. A binary variable was created representing current drug use with “no” representing “never used” (61%), “I just tried it” (17%), and “I used to use it but I don't anymore” (8%), and “yes” representing “I use it occasionally” (7%), “I only use it on weekends” (1%), and “I use it every day or almost every day” (5%).

#### Mediators: Police arrest (age 18), gang membership (age 18–22), and school noncompletion (22 years)

Police arrest was assessed by self‐report at the age of 18, asking if the young person had ever been arrested or detained (“yes” if ever arrested or detained and “no” otherwise). Four percent of young people had been arrested or detained by the police by age 18 years.

Gang membership was assessed by self‐report at ages 18 and 22 years with questions asking whether the participant had been a gang member in the previous year. Both time points were combined into a binary variable representing gang membership at either time point due to the low prevalence of gang membership (approximately 1% at both time points; 2% had been members of a gang in the previous year at age 18 or 22 years).

School noncompletion (assessed at age 22) was a dichotomous variable indicating if the young person has finished high school (passed all school grades) by age 22 (“yes” if not completed school and “no” if completed). Forty‐three percent of young people had not completed high school (passed all grades) by age 22. In Brazil, young people typically finish school by age 17, assuming they do not fail any grades; however, a student may continue studying into adulthood until they complete all grades (Martins et al., 2022).

#### Baseline and intermediate confounders

Baseline confounders and intermediate confounders were drawn from five domains (sociodemographic, individual, peer, family, and community) that have previously been identified as key factors influencing a young person's behavior (see Appendix S1). Baseline confounders included sex, a score of sociodemographic (e.g., low maternal education) and health (e.g., mother smoking in pregnancy) risk factors for conduct problems (assessed by maternal report or observation during the perinatal period), maternal depression, the quality of the father–child relationship, the quality of the mother–child relationship, parental alcohol consumption, parental smoking status, parental separation, and neighborhood safety (all assessed at child age 11 years). Intermediate confounders included peer deviance (assessed at child age 11 years), frequency of cigarette smoking and alcohol consumption, and peer drug use (all assessed at child age 15 years).

In secondary analyses, hyperactivity problems (at age 11 years) were considered as an additional baseline confounder and emotional problems (at age 15 years) were considered an additional intermediate confounder.

### Statistical analyses

**FIGURE 1:**
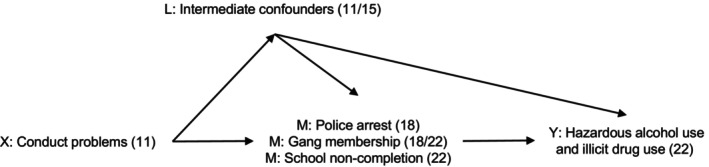
Directed Acyclic Graph showing hypothesized causal pathways between conduct problems and substance use (hazardous alcohol use and illicit drug use) through mediating “snares” (police arrest, gang membership, and school noncompletion). Baseline confounders (not shown on figure but assumed to confound all paths) included sex, sociodemographic risk factors, and health risk factors (assessed perinatally) and maternal depression, father–child relationship, mother–child relationship, parental alcohol consumption, parental smoking, parental separation, neighborhood safety, and hyperactivity problems (assessed at age 11); intermediate confounders included peer deviance (assessed at age 11) and frequency of cigarette smoking, frequency of alcohol consumption, peer drug use, and emotional problems (assessed at age 15); hyperactivity problems were considered a baseline confounder in secondary analyses, and emotional problems were considered an intermediate confounder in secondary analyses as these constructs represent comorbidity with conduct problems and the causal direction between them is less clear compared to other baseline/intermediate confounders.

Prior to conducting any statistical analysis, we constructed a Directed Acyclic Graph (DAG) to present hypothesized causal pathways based on previously published articles (Figure 1). All statistical analyses were carried out using Stata version 17 (StataCorp, 2021). First, we performed descriptive statistics to report characteristics of the population for the whole sample. Second, we conducted unadjusted and adjusted logistic regressions in stages to examine exposure–mediator and mediator–outcome associations. Third, we performed a counterfactual approach to mediation using the parametric g‐computation formula to estimate indirect effects using binary mediators and common binary outcomes, and incorporate intermediate confounders (De Stavola et al., 2015). The package ‐gformula‐ in Stata (Daniel et al., 2011) was used to estimate the total causal effect, natural direct effect and natural indirect effect via all three mediators simultaneously. All mediators were included simultaneously as we did not consider them to be independent. However, given that we were not able to confidently specify a causal direction between them, we were only able to estimate one indirect effect via all three mediators together. ‐gformula‐ uses Monte Carlo simulations to simulate the mediator, outcome, and intermediate confounders under each hypothetical “counter to the fact” scenario. The total causal effect represents the difference in the outcome comparing if everybody had been exposed to high conduct problems versus everyone having low conduct problems. The natural direct effect represents the direct (unmediated) effect of conduct problems on the outcome when the mediator takes the value it would have under conditions of absence of the exposure (X = 0). The natural direct effect is thus estimated as the effect of exposure X = 1 (high conduct problems) versus exposure X = 0 (low conduct problems) on outcome Y (e.g., hazardous alcohol use) if the mediator **M** (e.g., police arrest, gang membership, school noncompletion) were exactly that which arises under condition X = 0. The natural direct effect is useful to understand how much of the effect of conduct problems on the outcome (e.g., hazardous alcohol use) does not operate via changing the mediator (e.g. police arrest, gang membership, school noncompletion). This effect may not be truly direct, but via other variables on the causal pathway between the exposure and outcome. The natural indirect effect captures the effect of conduct problems on the outcome operating by changing the mediator. The natural indirect effect is modeled as the effect on the outcome Y (e.g., hazardous alcohol use) if the exposure were fixed at X = 1 and mediator **M** (e.g., police arrest, gang membership, and school noncompletion) were changed from its value if X = 0 to its value if X = 1.

Standard errors were estimated using 50 bootstrap samples and normal‐based 95% confidence intervals were calculated. More detail on the statistical analyses can be found in Appendix S2.

### Sensitivity analyses

First, we reran the mediation models making the assumption that police arrest (by age 18 years) and gang membership (at age 18 or 22 years) caused school noncompletion (by age 22 years), that is, we performed a single mediator model for school noncompletion with police arrest and gang membership as additional intermediate confounders. Although, the hypothesized mediators likely have bidirectional relationships between them, we made the a priori decision that the most likely causal ordering would be gang membership and police arrest leading to school noncompletion. This was based on previous literature (Connolly & Jackson, 2019; Gilman et al., 2014; Hirschfield, 2009; Kirk & Sampson, 2013) and the temporal ordering of measures. This assumption allowed us to assess the specific indirect effect via school noncompletion after accounting for gang membership and police arrest. Second, we reran main analyses using complete case rather than imputed data. Although we believe the results using multiple imputation will have greater power and be more robust than complete case analysis (potentially correcting bias from selective attrition under the assumption of missing‐at‐random), it is best practice to report both sets of results, discussing any differences between them (Lee et al., 2021). Finally, we examined the AUDIT subscales, alcohol consumption and alcohol problems, as alternative numeric outcomes. Using these subscales will both increase power and allow us to examine whether differences emerge comparing a more normative, common outcome in early adulthood (heavy consumption) to a more severe, rarer outcome (negative consequences experienced when drinking alcohol).

### Missing data

Of the 5249 newborns included in the cohort, 45% (*N* = 2342) had complete data on all analysis variables (Figure S1 shows a flow chart of retention). Table S1 provides a comparison of the complete case sample to the sample with missing information in at least one of the analysis variables (*n* = 2907) on the exposure and baseline confounders. As shown in Table S1, high conduct problems at age 11 were associated with missingness, as was male sex, a higher sociodemographic and health risk score, higher maternal depression symptoms, no parental separation, no parental alcohol use, poorer father–child and mother–child relationships, and higher hyperactivity problems.

To increase sample size, we performed multivariate imputation by chained equations to impute missing data on all variables, across all assessments up to our starting sample (those that provided data on alcohol use and drug use from at least one time point between age 11 and age 22 years; *N* = 4599). This starting sample was chosen to ensure we had strong auxiliary variables available to impute substance use at age 22 years. We generated 40 imputed data sets. For a detailed description of multiple imputation, see (Appendix S3: Table S2). Imputation and subsequent analysis code for the mediation models can be found on GitHub: https://github.com/gemmahammerton/gformula_1993_Pelotas. All analyses in the main text were performed on imputed data.

## RESULTS

### Descriptive statistics

Of 4599 children included in the imputed analyses, 51% were female, 77% had White mothers, and 23% had Black or mixed race mothers. Table 1 shows descriptive statistics for all analysis variables for the whole sample, using both imputed (*N* = 4599) and complete case data (*N* = 2342). Based on imputed data, 31% of children at age 11 years had high conduct problems, 4% of young people had been arrested or detained by the police by age 18 years, 2% had been members of a gang in the previous year at age 18 or 22 years, and 43% had not completed high school (passed all school grades) by age 22 years. Twenty‐two percent of young people reported hazardous alcohol consumption at age 22 years, and 14% reported current use of illicit drugs.

**TABLE 1:** Descriptive statistics for analysis variables for the whole sample using imputed and complete case data.

Variable	Imputed data (*N* = 4599)	Complete case data (*N* = 2342)
	%/Mean (95% CI)	%/Mean (95% CI)
Exposure (age 11)		
Conduct problems (high; 4+)	31% (29%–32%)	27% (26%–29%)
Mediators (age 18–22)		
Police arrest (yes)	4% (3%–4%)	3% (2%–3%)
Gang membership (yes)	2% (2%–3%)	1% (1%–2%)
School noncompletion (yes)	43% (42%–45%)	36% (34%–38%)
Outcomes (age 22)		
Hazardous alcohol use	22% (21%–24%)	21% (19%–22%)
Illicit drug use	14% (13%–15%)	13% (12%–14%)
Baseline confounders (perinatal)		
Female sex	51% (49%–52%)	54% (52%–56%)
Sociodemographic risk score (0–5)	0.90 (0.87–0.93)	0.78 (0.74–0.82)
Health risk score (0–6)	1.56 (1.52–1.59)	1.51 (1.47–1.56)
Baseline confounders (age 11)		
Maternal depression (0–20)	5.69 (5.56–5.82)	5.47 (5.30–5.65)
Fear of the neighborhood (yes)	12% (11%–13%)	11% (10%–13%)
Parental separation (yes)	57% (56%–59%)	54% (52%–56%)
Parental smoking (yes)	73% (72%–74%)	72% (70%–74%)
Parental alcohol use (yes)	68% (66%–69%)	69% (67%–71%)
Father–child relationship (1 (worst)–5)	4.10 (4.06–4.13)	4.15 (4.10–4.20)
Mother–child relationship (1 (worst)–5)	4.43 (4.40–4.46)	4.49 (4.46–4.53)
Comorbidity (age 11)		
Hyperactivity problems (0–10)	4.30 (4.21–4.39)	4.10 (3.97–4.22)
Intermediate confounders (age 11–15)		
Peer deviance (yes)	47% (45%–48%)	45% (43%–47%)
Frequency of cigarette smoking (1–5 days in last month)	6% (5%–7%)	5% (4%–5%)
Frequency of alcohol use (1–5 days in last month)	25% (24%–26%)	24% (23%–26%)
Peer drug use (yes)	13% (12%–14%)	12% (11%–14%)
Comorbidity (age 15)		
Emotional problems (0–10)	3.93 (3.84–4.01)	3.82 (3.71–3.92)

### Associations between conduct problems (exposure) and police arrest, gang membership and school noncompletion (mediators)

**FIGURE 2:**
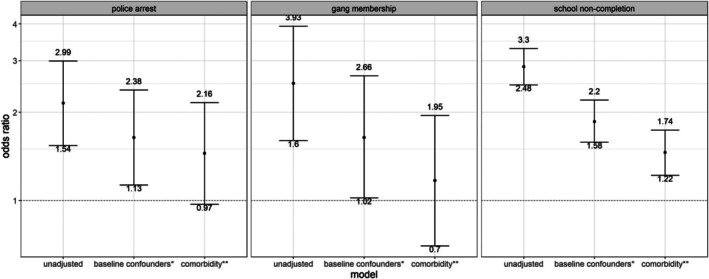
Associations between the exposure (conduct problems at age 11 years) and the mediators (police arrest, gang membership, and school noncompletion between age 18 and 22 years) using imputed data; *N* = 4599. Figure showing odds ratio and 95% confidence interval; *adjusted for baseline confounders including sex, score of sociodemographic risk factors, score of health risk factors (all measured perinatally), maternal depression, father/child relationship, mother/child relationship, parental alcohol consumption, parental smoking, parental separation, neighborhood safety (all measured at age 11); **adjusted for baseline confounders listed above, and additionally adjusted for hyperactivity problems at age 11.

Figure 2 shows associations between the exposure (high conduct problems) and the mediators (police arrest, gang membership, and school noncompletion) before and after adjusting for confounders. After adjusting for baseline confounders and hyperactivity problems, those with high childhood conduct problems had slightly higher odds of being arrested by the police by age 18 (OR [95% CI] = 1.45 [0.97, 2.16]) and not completing school by age 22 (OR [95% CI] = 1.46 [1.22, 1.74]) compared to those with low childhood conduct problems. There was no evidence of an association between conduct problems and gang membership after adjusting for baseline confounders and hyperactivity problems (OR [95% CI] = 1.17 [0.70, 1.95]).

### Associations between police arrest, gang membership, and school noncompletion (mediators) and hazardous alcohol use and illicit drug use (outcomes)

Univariable associations for baseline confounders with mediators and outcomes are shown in Table S3 and univariable associations for intermediate confounders are shown in Table S4.

Table 2 shows associations between the mediators and the outcomes before and after adjusting for confounders (including hyperactivity and emotional problems). After adjusting for all confounders, those who had been arrested (OR [95% CI] = 1.60 [1.08, 2.38]) and those in a gang (OR [95% CI] = 1.88 [1.07, 3.30]) had slightly higher odds of hazardous alcohol use at age 22. There was no evidence of an association between school noncompletion and hazardous alcohol use after adjusting for confounders (OR [95% CI] = 1.10 [0.90, 1.33]). After adjusting for confounders, those who had been arrested (OR [95% CI] = 3.84 [2.46, 5.99]) and those in a gang (OR [95% CI] = 7.78 [4.30, 14.10]) had higher odds of illicit drug use at age 22. There was no evidence of an association between school noncompletion and illicit drug use after adjusting for confounders (OR [95% CI] = 1.17 [0.92, 1.49]).

**TABLE 2:** Associations between the mediators (police arrest, gang membership, and school noncompletion between age 18 and 22 years) and the outcomes (hazardous alcohol use and illicit drug use at age 22 years) using imputed data; *N* = 4599.

OR (95% CI); *p* value	Unadjusted	Adjusted for baseline and intermediate confounders^a^	Additionally adjusted for comordidity^b^	Additionally adjusted for all mediators
Hazardous alcohol use				
Police arrest	2.70 (1.81, 4.04); <0.001	1.63 (1.09, 2.42); 0.016	1.60 (1.08, 2.38); 0.019	1.50 (1.00, 2.25); 0.052
Gang membership	2.64 (1.61, 4.32); <0.001	1.97 (1.13, 3.45); 0.017	1.88 (1.07, 3.30); 0.027	1.74 (0.98, 3.10); 0.060
School non‐completion	1.28 (1.09, 1.50); 0.002	1.14 (0.95, 1.38); 0.167	1.10 (0.90, 1.33); 0.350	1.07 (0.88, 1.29); 0.520
Illicit drug use				
Police arrest	6.38 (4.32, 9.41); <0.001	3.86 (2.48, 6.01); <0.001	3.84 (2.46, 5.99); <0.001	3.29 (2.05, 5.27); <0.001
Gang membership	9.75 (5.75, 16.53); <0.001	7.81 (4.32, 14.10); <0.001	7.78 (4.30, 14.10); <0.001	6.79 (3.64, 12.64); <0.001
School non‐completion	1.28 (1.05, 1.56); 0.015	1.17 (0.92, 1.48); 0.201	1.17 (0.92, 1.49); 0.203	1.04 (0.81, 1.34); 0.749

Abbreviations: 95% CI, 95% confidence interval; OR, odds ratio.

^a^ Adjusted for high conduct problems at age 11 and baseline confounders including sex, score of sociodemographic risk factors, score of health risk factors (all measured perinatally), maternal depression, father/child relationship, mother/child relationship, parental alcohol consumption, parental smoking, parental separation, neighborhood safety (all measured at age 11), and intermediate confounders including peer deviance (at age 11), frequency of cigarette smoking (at age 15), frequency of alcohol drinking (at age 15), peer drug use (at age 15).

^b^ Adjusted for baseline and intermediate confounders listed above, and additionally adjusted for hyperactivity problems at age 11 as a baseline confounder and emotional problems at age 15 as an intermediate confounder.

### Indirect effects of conduct problems (exposure) on hazardous alcohol use and illicit drug use (outcomes) via police arrest, gang membership, and school noncompletion (mediators)

Table 3 shows the total causal effect, natural direct effect and natural indirect effect of conduct problems on hazardous alcohol use, and illicit drug use via all three mediators simultaneously. After adjusting for baseline and intermediate confounders, there was weak evidence for a small total effect of conduct problems on hazardous alcohol use (OR [95% CI] = 1.18 [1.00, 1.39]), representing an 18% increase in the odds of hazardous alcohol use if everybody had been exposed to high conduct problems versus everyone having low conduct problems.

**TABLE 3:** Mediation models including all mediators (police arrest, gang membership, and school noncompletion) simultaneously using imputed data; *N* = 4599.

OR (95% CI)	Unadjusted	Adjusted for baseline and intermediate confounders^a^	Additionally adjusted for comorbidity^b^
Hazardous alcohol use			
TCE	1.29 (1.09, 1.52)	1.18 (1.00, 1.39)	1.02 (0.84, 1.24)
NDE	1.19 (0.99, 1.42)	1.14 (0.97, 1.35)	1.00 (0.82, 1.22)
NIE	1.09 (1.04, 1.14)	1.03 (1.00, 1.06)	1.01 (0.99, 1.04)
PM	33%	20%	n/a
Illicit drug use			
TCE	1.20 (0.99, 1.46)	1.05 (0.86, 1.30)	1.01 (0.79, 1.31)
NDE	1.04 (0.84, 1.27)	0.97 (0.79, 1.20)	0.98 (0.77, 1.26)
NIE	1.16 (1.06, 1.27)	1.08 (1.01, 1.17)	1.03 (0.96, 1.10)
PM	81%	n/a	n/a

Abbreviations: 95% CI, 95% Confidence Interval; n/a, not calculated due to inconsistent mediation or a very small total effect; NDE, Natural Direct Effect; NIE, Natural Indirect Effect; OR, odds ratio; PM, proportion mediated; TCE, total causal effect.

^a^ Adjusted for baseline confounders including sex, score of sociodemographic risk factors, score of health risk factors (all measured perinatally), maternal depression, father/child relationship, mother/child relationship, parental alcohol consumption, parental smoking, parental separation, neighborhood safety (all measured at age 11), and intermediate confounders including peer deviance (at age 11), frequency of cigarette smoking (at age 15), frequency of alcohol drinking (at age 15), peer drug use (at age 15).

^b^ Adjusted for baseline and intermediate confounders listed above, and additionally adjusted for hyperactivity problems at age 11 as a baseline confounder and emotional problems at age 15 as an intermediate confounder.

There was little evidence for a total effect of conduct problems on illicit drug use (OR [95% CI] = 1.05 [0.86, 1.30]). There was weak evidence for a small natural indirect effect of conduct problems on hazardous alcohol use (OR [95% CI] = 1.03 [1.00, 1.06]) and illicit drug use (OR [95% CI] = 1.08 [1.01, 1.17]) via all three mediators. These results represent a 3% and 8% increase in the odds of hazardous alcohol use and illicit drug use, respectively, considering if everybody had been exposed to high conduct problems (X = 1) and the mediators were changed from their level under conditions of being unexposed to high conduct problems (X = 0) to their level if exposed (X = 1).

After additionally adjusting for hyperactivity (at age 11) as a baseline confounder and emotional problems (at age 15) as an intermediate confounder, all effects weakened. There was no longer evidence for a total effect of conduct problems on hazardous alcohol use (OR [95% CI] = 1.02 [0.84, 1.24]), or a natural indirect effect of conduct problems on hazardous alcohol use (OR [95% CI] = 1.01 [0.99, 1.04]) or illicit drug use (OR [95% CI] = 1.03 [0.96, 1.10]) via all three mediators.

### Sensitivity analyses

Table S5 shows the total causal effect, natural direct effect and natural indirect effect of conduct problems on hazardous alcohol use and illicit drug use via school noncompletion, treating police arrest and gang membership as intermediate confounders. There was no evidence for an indirect effect via school noncompletion, for either hazardous alcohol use or illicit drug use after adjusting for confounders.

Tables S6–S8 show results for analyses using complete case data (*N* = 2342). In general, odds ratios were slightly stronger but confidence intervals were wider using the complete case sample; however, conclusions were unchanged.

Table S9 shows associations between the mediators and the AUDIT subscales (alcohol consumption and alcohol problems) before and after adjusting for confounders (including hyperactivity and emotional problems). After adjusting for all confounders, those who had been arrested (b [95% CI] = 0.86 [0.36, 1.36]) and those in a gang (b [95% CI] = 0.79 [0.10, 1.49]) had higher levels of alcohol consumption at age 22. There was no evidence of an association between school noncompletion and alcohol consumption after adjusting for confounders (b [95% CI] = 0.11 [−0.08, 0.30]). Similarly, after adjusting for all confounders, those who had been arrested [b (95% CI) = 0.69 (0.06, 1.32)] and those in a gang [b (95% CI) = 0.82 (0.05, 1.58)] had higher levels of alcohol problems at age 22. Again, there was no evidence of an association between school noncompletion and alcohol problems after adjusting for confounders (b [95% CI] = 0.11 [−0.12, 0.35]).

Table S10 shows the total causal effect, natural direct effect and natural indirect effect of conduct problems on alcohol consumption and alcohol problems via all three mediators simultaneously. After adjusting for baseline and intermediate confounders, there was weak evidence for a small total effect of conduct problems on alcohol consumption (b [95% CI] = 0.20 [0.01, 0.39]) and alcohol problems (b [95% CI] = 0.22 [−0.01, 0.44]). There was weak evidence for a small natural indirect effect of conduct problems on alcohol consumption (b [95% CI] = 0.03 [0.001, 0.06]) and alcohol problems (b [95% CI] = 0.03 [−0.004, 0.07]) via all three mediators. After additionally adjusting for hyperactivity and emotional problems, all effects weakened. There was no longer evidence for a total effect of conduct problems on alcohol consumption (b [95% CI] = 0.09 [−0.14, 0.32]) or alcohol problems (b [95% CI] = 0.10 [−0.16, 0.36]), or a natural indirect effect of conduct problems on alcohol consumption (b [95% CI] = 0.01 [−0.01, 0.04]) or alcohol problems (b [95% CI] = 0.01 [−0.01, 0.04]) via all three mediators.

## DISCUSSION

Using a large, population‐based birth cohort in Brazil, we found some evidence to support our first hypothesis. There was weak evidence that high childhood conduct problems were associated with being arrested by the police by age 18 years and slightly stronger evidence for an association with not completing school by age 22 years after adjusting for potential confounders, including hyperactivity problems. We found an association between childhood conduct problems and gang membership in late adolescence, but this weakened substantially after accounting for hyperactivity problems. We also found some evidence to support our second hypothesis. Being arrested by the police and gang membership were strongly associated with illicit drug use and weakly associated with hazardous alcohol use at age 22 years, after adjusting for confounders. There was no association between school noncompletion and either outcome after adjusting for confounders. Contrary to our third hypothesis, the indirect effects of conduct problems on substance use via police arrest, gang membership, school noncompletion were very small, particularly after adjusting for hyperactivity and emotional problems.

We found that childhood conduct problems were associated with not completing school by age 22 years after adjusting for confounders, including childhood hyperactivity problems. This finding partially supports a previous study using a school‐based community cohort of Brazilian children and adolescents (aged 6–14 years) which found that externalizing disorders (including attention deficit and hyperactivity, conduct and oppositional‐defiant disorders) were associated with grade repetition and age‐grade distortion 3 years later (Hoffmann et al., 2021), although not with school dropout. This finding also supports evidence from HIC showing an association between childhood conduct problems and school noncompletion (Colman et al., 2009; Erskine et al., 2016; Gubbels et al., 2019; Kulkarni et al., 2021; Lau et al., 2023). Contrary to the conclusion in a recent systematic review (Kulkarni et al., 2021), we found an association between conduct problems and school noncompletion after adjusting for hyperactivity problems. However, although high childhood conduct problems were associated with both police arrest and gang membership after adjusting for confounders, these associations weakened after adjusting for childhood hyperactivity problems, particularly for gang membership. This supports previous reviews of the literature which highlight the importance of risk‐taking, impulsivity, and low self‐control as risk factors for joining a gang (Higginson et al., 2018; Howell & Egley, 2005; Maxson, 2011). Any one risk factor in isolation is unlikely to be sufficient to identify young people at high or low risk of joining a gang; alternatively, the total accumulation of risk factors or exposure to risk factors across multiple domains is important (Hill et al., 1999; Howell & Egley, 2005; Thornberry et al., 1993).

In the current study, there was no association between school noncompletion and either hazardous alcohol use or illicit drug use after adjusting for confounders. This is in contrast to a study of American adolescents which found an association between school failure and alcohol use 1 year later (Crosnoe, 2006). The lack of association in the current study could be explained by the wide range of confounders adjusted for. Alternatively, it could be due to the age of the young people, given that those who have completed school may be attending university at age 22, where levels of substance use are generally high. Despite the lack of association between school noncompletion and substance use, it is still likely that school noncompletion is an important snare in Brazil, that may trap those with childhood conduct problems into involvement with violent and nonviolent crime in early adulthood (Martins et al., 2022).

In contrast to the findings for school noncompletion, we found that being arrested by the police and gang membership were weakly associated with hazardous alcohol use, and strongly associated with illicit drug use at age 22 years, even after adjusting for confounders. This supports results from previous studies that found associations between gang membership (Gordon et al., 2004; Harris et al., 2013; Higginson et al., 2018; Swahn et al., 2010) and police arrest (Newman & Crowell, 2023) with problematic substance use.

Due to the longitudinal design and wealth of data in the 1993 Pelotas Birth Cohort, we were able to examine mediators of the association between conduct problems at age 11 and substance use over 10 years later, while adjusting for potential baseline and intermediate confounders across multiple domains, including sociodemographic, individual, peer, family, and the community. However, our results need to be interpreted in light of several limitations. First, in our population‐based sample, few people joined a gang or were arrested by the police (2% and 4%, respectively); therefore, the weak evidence for associations could be explained by a lack of power and other factors more common among children with conduct problems might be more important mediators for the relationship between conduct problems and substance use. For example, studies using data in HIC have identified deviant peer influences (Scalco et al., 2014; Van Eck et al., 2014), parenting and family relationships (Krohn et al., 2019), and patterns of externalizing behaviors into adolescence (Englund et al., 2008; Hammerton et al., 2020; Wertz et al., 2018) as mediators of the relationship between conduct problems and substance use. Additionally, there is evidence from U.S. interventions (Godwin et al., 2020; Sorensen et al., 2016) that suggests that intervening to improve interpersonal or intrapersonal skills can disrupt the relationship between conduct problems and substance use (and other negative outcomes such as criminal behavior). Therefore, mediators such as prosocial or emotional recognition skills will be important for future studies to explore, particularly in LMIC.

Second, even though we adjusted for a wide range of confounders, there might be some residual confounding present in our analysis due to either unmeasured confounders (such as genetic risk) or confounders measured with error. Third, as with most cohort studies, there was selective attrition over time, with missingness associated with conduct and hyperactivity problems, alongside sociodemographic and family‐based confounders. However, we used multiple imputation to address missing data incorporating a wide range of strong auxiliary variables to make the missing‐at‐random assumption plausible and conclusions using complete case and imputed data were similar.

Fourth, although the exposure and outcomes were assessed using established scales that have been validated in Brazil, each mediator was assessed using a single item. Additionally, the measure of school noncompletion captures both dropping out of school and grade repetition. It may be that school dropout is a more important risk factor for substance use than grade repetition, but we were not able to distinguish between these related constructs. Fifth, we have focused on substance use in early adulthood when alcohol consumption is fairly normative. Although we categorized alcohol use into “hazardous levels” (which included 22% of the sample) and found similar results using an alcohol problems subscale, it may be that our hypothesized “snares” have stronger effects on substance use at other ages, or more severe problematic behaviors in early adulthood.

Finally, although there is consistent evidence for a relationship between conduct problems and substance use from longitudinal studies, even adjusting for a range of relevant confounders (Bevilacqua et al., 2018; Fergusson et al., 2007; Grant et al., 2015; Hammerton et al., 2019), it is possible that this is explained by unmeasured confounders and is not therefore causal. In the current study, there is little evidence for an association between conduct problems and substance use, particularly for illicit drug use, after adjusting for confounders. Further research, particularly using data from LMIC, should use research designs that permit stronger causal inference to establish whether the relationships found between conduct problems and substance use in longitudinal studies are causal.

## CONCLUSION

The findings from the current study highlight the importance of school professionals being aware of the risk for school noncompletion for those with childhood conduct problems and collaboration between education and health sectors in Brazil. Early intervention to support those with childhood conduct problems to continue with their education is a priority. However, it is also important to consider that many behavioral problems have to do with not adapting to the school system, which is not a problem in itself for the child or adolescent, but our inability to think about education in other ways.

The findings also highlight the importance of early intervention and treatment for hyperactivity (in addition to conduct problems) to prevent long‐term negative outcomes such as gang membership, criminal activity, and substance use in Brazil. Given the high rates of comorbidity of conduct and hyperactivity problems, it is difficult to examine the independent effects and preventing or treating both is a priority (Erskine et al., 2016; Fergusson et al., 2007). Finally, programmes and policies designed to reduce substance use in Brazil should focus on young people involved in gangs, and in the criminal justice system.

Although there is some evidence in LMIC for short‐term effects of early childhood interventions, particularly those designed to improve parent–child relationships (Backhaus et al., 2023; Burkey et al., 2018; Knerr et al., 2013), or focusing on improving social and emotional development (Baker‐Henningham, 2014; Burkey et al., 2018), evidence for specific improvements in childhood behavioral problems has been more limited (Francis & Baker‐Henningham, 2021; Lachman et al., 2017). A recent randomized controlled trial in Pelotas, Brazil found that two parenting programmes had small effects on parenting but did not reduce child aggression or several other risk/protective factors for violence (Murray et al., 2024). Additionally, there was a recent evaluation of the “Pelotas Pact for Peace” programme, whose goal was to reduce violence and crime in Pelotas (Degli Esposti et al., 2023). The programme led to an overall reduction in homicide and robbery in Pelotas, but there was no reduction in nonviolent property crime, violence against women or school dropout (Degli Esposti et al., 2023). In the current study, we found little evidence for an indirect effect of childhood conduct problems on substance use in early adulthood via school noncompletion, gang membership, and police arrest after accounting for potential confounders. This might be because the causes of problematic substance use in children with conduct problems operate early in life (Lahey, 2015). Alternatively, there may be other mediators, such as exposure to trauma or the influence of deviant peers, which might have a stronger influence on later substance use (Grummitt et al., 2021). Future research is needed to examine the indirect effects of childhood hyperactivity problems on later substance use in Brazil and to examine the potential snares that may trap children with conduct problems into other negative long‐term outcomes such as criminal behavior and unemployment.

## AUTHOR CONTRIBUTIONS

All authors have made a substantial contribution to the article. FS, JH, JM, and GH contributed to the concept or design of the article; FCW, HG, AMBM, JM, and GH contributed to the acquisition of data; FS and GH contributed to the analysis of data; FS, JH, MH, FCW, HG, AMBM, JM, and GH contributed to the interpretation of data; FS and GH contributed to writing the manuscript; FS, JH, MH, FCW, HG, AMBM, JM, and GH contributed to revising the manuscript; and JH, JM, and GH contributed to supervision.

## FUNDING INFORMATION

This research was funded by a Sir Henry Wellcome Postdoctoral Fellowship (209138/Z/17/Z) awarded to Dr. Hammerton. Drs. Hammerton and Heron are members of the MRC Integrative Epidemiology Unit at the University of Bristol (MC_UU_00011/7). This research was funded in whole, or in part, by the Wellcome Trust (209138/Z/17/Z; 210735_A_18_Z). For the purpose of Open Access, the authors have applied a CC BY public copyright license to any Author Accepted Manuscript version arising from this submission. Professor Murray is supported by a Wellcome Trust Investigator Award (210735_A_18_Z). Professors Menezes, Gonçalves, Wehrmeister, and Murray are supported by the Brazilian National Research Council (CNPq). The funding sources had no role in the study design, analysis of data, interpretation of results, or writing of the report.

## CONFLICT OF INTEREST STATEMENT

None declared.

## ETHICS STATEMENT

In all phases of the study (1993, 2004, 2008, 2011, 2015), ethical approval was obtained from the Federal University of Pelotas Medical School Ethics Committee.

## PATIENT CONSENT STATEMENT

After being informed of the details of the study, participants signed a term of informed consent. Full informed consent was also provided by parents (if the subject was aged under 18 years) or by cohort members in the later phases of the study. The authors assert that all procedures contributing to this work comply with the ethical standards of the relevant national and institutional committees on human experimentation and with the Helsinki Declaration of 1975, as revised in 2008.

## Supporting information


Appendix S1.

## Data Availability

Data from the 1993 Pelotas Birth Cohort are available to researchers upon request to the specific cohort committee. Further details are provided on the Cohort website: https://epidemio‐ufpel.org.br/coorte‐1993/. The code used in this manuscript can be found on GitHub: https://github.com/gemmahammerton/gformula_1993_Pelotas.
