# Supplementary material for: Childhood conduct problems, potential snares in adolescence, and problematic substance use in Brazil
Source: J Res Adolesc. 2025 Nov 19;35(4):e70099. doi: 10.1111/jora.70099 (PMC12630428; doi:10.1111/jora.70099)
Supplement: Supplementary file 1 — Appendix S1. [file JORA-35-0-s001.docx]

**Supplementary material**

[**Appendix S1.** Details on the baseline and intermediate confounders 2](#_Toc205904661)

[**Appendix S2.** Further details on the statistical analyses including i) logistic regression models and ii) the counterfactual approach to mediation 5](#_Toc205904662)

[**Figure S1.** Flow chart of retention in the 1993 Pelotas Birth Cohort 8](#_Toc205904663)

[**Table S1.** Comparison of complete cases (n = 2,342) and those with missing information in at least one of the analysis variables (n = 2,907) 9](#_Toc205904664)

[**Appendix S3.** Details on multiple imputation 10](#_Toc205904665)

[**Table S2.** Summary of missing data for analysis and auxiliary variables 12](#_Toc205904666)

[**Table S3.** Univariable associations for baseline confounders with mediators and outcomes using imputed data; *N* = 4,599 15](#_Toc205904667)

[**Table S4.** Univariable associations for intermediate confounders with mediators and outcomes using imputed data; *N* = 4,599 17](#_Toc205904668)

[**Table S5.** Mediation models for school non-completion with police arrest and gang membership treated as intermediate confounders using imputed data; *N* = 4,599 18](#_Toc205904669)

[**Table S6.** Associations between the exposure (conduct problems at age 11 years) and the mediators (police arrest, gang membership, and school non-completion between age 18 and 22 years) using complete case data; N = 2*,*342 19](#_Toc205904670)

[**Table S7.** Associations between the mediators (police arrest, gang membership, and school non-completion between age 18 and 22 years) and the outcomes (hazardous alcohol use and illicit drug use at age 22 years) using complete case data; N = 2*,*342 20](#_Toc205904671)

[**Table S8.** Mediation models including all mediators (police arrest, gang membership, and school non-completion) simultaneously using complete case data; N = 2*,*342 21](#_Toc205904672)

[**Table S9.** Associations between the mediators (police arrest, gang membership, and school non-completion between age 18 and 22 years) and the AUDIT subscales (alcohol consumption and alcohol problems at age 22 years) using imputed data; *N* = 4,599 22](#_Toc205904673)

[**Table S10.** Mediation models including all mediators (police arrest, gang membership, and school non-completion) simultaneously using imputed data; *N* = 4,599 23](#_Toc205904674)

[**Supplementary references** 24](#_Toc205904675)

# **Appendix S1.** Details on the baseline and intermediate confounders

*Baseline and intermediate confounders*

Baseline confounders included sex, a score of sociodemographic and health risk factors for conduct problems, maternal depression, the quality of the father-child and mother-child relationship, parental alcohol consumption, parental smoking status, parental separation, and neighbourhood safety. These were chosen based on evidence from previous literature of associations with conduct problems ^1–4^, the potential snares ^2,4–8^, and substance use ^9–13^.

Sociodemographic and health risk factors were assessed by maternal report during the perinatal period. Sociodemographic factors included maternal age (< 20 years/≥20 years), low maternal education (yes/no; referring to 0–8 vs≥ 9 years of schooling), marital status (single mother/ with partner), three or more siblings (yes/no) and family income (lowest quintile/ second-fifth quintiles). The cumulative number of sociodemographic risk factors was summed, up to five, for each child as has been done previously ^2^, with five indicating the highest risk for conduct problems and substance use. Health risk factors included unplanned pregnancy (yes/no), mother ever smoked in pregnancy (yes/no), mother used alcohol in pregnancy (yes/no), maternal urinary infection during pregnancy (yes/no), intrauterine growth restriction (yes/no; referring to < 10th percentile/≥ 10th percentile for gestational age and sex, according to the reference curve developed by Kramer and colleagues ^14^ and premature birth < 37 weeks (yes/no). Again, the cumulative number of health risk factors was summed, up to six, for each child as has been done previously ^2^.

All other baseline confounders were assessed when the child was 11 years old. Maternal depression was assessed with mothers using the Self Report Questionnaire, which has been validated in Brazil ^15^. The quality of the mother-child and father-child relationship was assessed with child-report questions including the following response options: bad, regular, good, very good, excellent. Parental alcohol consumption was assessed using two maternal-report questions, capturing whether she and/or her husband never, not currently or currently drink alcohol. Likewise, parental smoking was assessed using two maternal-report questions, one addressing the mother’s lifetime smoking status and one addressing the father’s lifetime smoking status (ever versus never). Parental separation was assessed using a maternal-report question about the child’s biological father living in the same household, and a child-report question about parental divorce. A binary variable was created, where either child-report or maternal-report of the father not living in the same household were classed as parental separation. Neighbourhood safety was assessed using a maternal-report question asking whether she was afraid of living in the neighbourhood (yes/no).

Intermediate confounders included peer deviance, frequency of cigarette smoking and alcohol consumption, and peer drug use. Intermediate confounders were chosen based on evidence that they confound the associations between the potential snares and substance use ^5–8,10,12,16,17^, and are also causally downstream from childhood conduct problems (our exposure) ^5,12,18–20^.

Peer deviance was assessed at age 11 by asking the young person’s mother if she considered her child’s friends as having a bad influence or being bad company (yes/no). Frequency of cigarette smoking and frequency of alcohol consumption were assessed at age 15 by child-report. The questionnaire item for smoking had five response categories: ‘Never smoked cigarettes, not smoked in the last month, 1 to 5 days, 6 to 9 days, ten or more days and every day in the last month’ and the questionnaire item for alcohol had five response categories: ‘Never drank alcohol, not drank alcohol in the last month, one to five days, six to nine days, ten or more days and every day in last month’. For both items, the last three response categories were combined to the category “current smoking” or “current drinking”, respectively, due to small cell counts. Peer drug use was assessed by child-report at age 15. A binary variable was created indicating if friends or anyone in the same class as the young person used cannabis, glue, solvents, or cocaine.

*Comorbidity: hyperactivity problems (age 11) and emotional problems (age 15)*

In secondary analyses, hyperactivity was considered as an additional baseline confounder based on evidence for associations with conduct problems ^1,3,4,21^, the potential snares ^6,7,20,21^, and substance use ^20,22–24^. Hyperactivity problems were assessed by maternal-report at child age 11 years using the five-item hyperactivity problems subscale from the Strengths and Difficulties Questionnaire (SDQ) ^25^. The five items in the questionnaire include ‘restless, overactive, cannot stay still for long’, ‘constantly fidgeting or squirming’, ‘easily distracted, concentration wanders’, ‘thinks things out before acting’ and ‘sees tasks through to the end, good attention span’. Responses for each item are ‘Not true’, ‘Somewhat true’ and ‘Certainly true’. The items ‘thinks things out’ and ‘sees tasks through’ were reverse coded. All items were summed together (range 0 to 10).

In secondary analyses, emotional problems were considered as an additional intermediate confounder given that they may be a precursor for the potential snares and substance use ^24,26–29^, and are also causally downstream from childhood conduct problems (our exposure) ^20,30^. Emotional problems were assessed by maternal-report at age 15 years using the five-item emotional problems subscale from the SDQ. The five items in the questionnaire include ‘often complains of headaches, stomach-aches or sickness’, ‘many worries, often seems worried’, ‘often unhappy, down-hearted or tearful’, ‘nervous or clingy in new situations, easily loses confidence’ and ‘many fears, easily scared’. Responses for each item are ‘Not true’, ‘Somewhat true’ and ‘Certainly true’. All items were summed together (range 0 to 10).

# **Appendix S2.** Further details on the statistical analyses including i) logistic regression models and ii) the counterfactual approach to mediation

*Logistic regression models*

We conducted unadjusted and adjusted logistic regressions in stages to examine exposure-mediator and mediator-outcome associations. For exposure-mediator regressions, we first adjusted for baseline confounders, followed by baseline confounders including hyperactivity problems. For mediator-outcome regressions, we first adjusted for baseline and intermediate confounders, followed by these confounders including hyperactivity and emotional problems, followed by a regression model simultaneously including all three mediators, and all confounders. All regression models were governed by the same Directed Acyclic Graph, hence for mediator-outcome regressions, the exposure (conduct problems) becomes another confounder. Caution is required when directly comparing across these logistic regression models, each with different sets of confounders, given that the non-collapsibility of the odds ratio with a common binary outcome (22% for hazardous alcohol use and 14% for illicit drug use) means that the difference between an unadjusted and adjusted odds ratio can be due to both confounder adjustment and non-collapsibility ^31^.

*Counterfactual mediation*

The counterfactual approach is based on conceptualising ‘potential outcomes’ for each individual [Y(*x*)] that would have been observed if particular conditions were met (i.e. had the exposure X been set to the value *x* through some intervention) – regardless of the conditions that were in fact met for each individual ^32^. In mediation analyses, four assumptions are made with respect to confounding. These include no unmeasured confounders for any of the paths (exposure-outcome, exposure-mediator and mediator-outcome) and no measured or unmeasured confounders for the association between mediator and outcome which lie on the causal pathway from the exposure. In the current study, we assume that the same set of baseline confounders (sex, a score of sociodemographic and health risk factors, maternal depression, the quality of the father-child and mother-child relationship, parental alcohol consumption, and smoking status, parental separation, neighbourhood safety, and hyperactivity problems) confound all paths, and these were assessed before or during the same assessment as the exposure.

The counterfactual approach to mediation using the parametric g-computation formula was performed to relax the assumption of no measured intermediate confounding and to include binary mediators and outcomes. This counterfactual approach simulates the mediator, outcome and intermediate confounders under each hypothetical intervention or “counter to the fact” scenario. Again, mediation models were performed in stages. First we performed unadjusted models. Next, we adjusted for baseline and intermediate confounders, and finally, we adjusted for all confounders including hyperactivity and emotional problems. Unlike the logistic regression models, -gformula- presents mediation effects as marginal odds ratios meaning that non-collapsibility of the odds ratio is no longer a consideration. We did not hypothesise an exposure-mediator interaction and therefore did not include it in our models. The proportion of the total causal effect that was mediated was calculated by dividing the log odds ratio for the natural indirect effect by the log odds ratio for the total causal effect, and multiplying by 100. We used a Monte Carlo sample size of 10,000 to minimise fluctuations in effect estimates, and a seed of 79.

The total causal effect (TCE) is the value the outcome would take if everybody had been exposed to high conduct problems versus everyone having low conduct problems. The natural direct effect (NDE) is the direct (unmediated) effect of the exposure on the outcome when the mediator takes the value it would take in the absence of the exposure. It is thus modelled as the direct effect of exposure X = 1 (high conduct problems) versus exposure X = 0 (low conduct problems) on outcome Y (e.g., hazardous alcohol use) if mediator M (e.g., gang membership) were set to whatever it would be for X = 0.

NDE = E[*Y*(1, *M*(0))] - E[*Y*(0, *M*(0))]

The natural indirect effect (NIE) captures the effect of the exposure on the outcome that operates by changing the mediator. It is thus modelled as the effect on outcome Y (e.g., hazardous alcohol use) if the exposure were fixed at X = 1 and mediator M (e.g., gang membership) were changed from the level it would take if X = 0 to the level it would take if X = 1.

NIE = E[*Y*(1, *M*(1))] - E[*Y*(1, *M*(0))]

# **Figure S1.** Flow chart of retention in the 1993 Pelotas Birth Cohort

Live births in Pelotas

*N* = 5,265

Data available on exposure (conduct problems)

*N* = 4,373

16 mothers could not be interviewed or refused to participate in the study

Newborns included in cohort

*N* = 5,249

Data available on alcohol and drug use from at least one assessment (age 11, 15, 18, 22)

*N* = 4,599

Imputed sample

Complete case sample

Data available on all analysis variables

*N* = 2,342

# **Table S1.** Comparison of complete cases (n = 2,342) and those with missing information in at least one of the analysis variables (n = 2,907)

|  | Proportion missingness | Complete cases  *n* = 2342 | | Cases with missing data  *n* = 2907 | | Univariable association with missingness | |  |
| --- | --- | --- | --- | --- | --- | --- | --- | --- |
| Variable | % | *n* | (%) or Mean (SD) | *n* | (%) or Mean (SD) | OR | (95% CI) | p-value |
| **Exposure (age 11)** |  |  |  |  |  |  |  |  |
| Conduct problems (high; 4+) | 16% | 640. | (27.33) | 733. | (35.53) | 1.47 | (1.30 - 1.67) | <0.0001 |
| **Baseline confounders (perinatal)** |  |  |  |  |  |  |  |  |
| Female sex | <1% | 1268. | (54.14) | 1377. | (47.38) | 0.76 | (0.68 - 0.85) | <0.0001 |
| Sociodemographic risk score (0 - 5) | 0% | 2342 | 0.78 (0.92) | 2907 | 1.03 (1.04) | 1.30 | (1.23 - 1.37) | <0.0001 |
| Health risk score (0 - 6) | 11% | 2342 | 1.51 (1.07) | 2330 | 1.59 (1.07) | 1.07 | (1.02 - 1.13) | 0.009 |
| **Baseline confounders (at age 11)** |  |  |  |  |  |  |  |  |
| Maternal depression (0 - 20) | 16% | 2342 | 5.47 (4.36) | 2060 | 6.01 (4.70) | 1.03 | (1.01 - 1.04) | <0.0001 |
| Fear of the neighbourhood (yes) | 16% | 267. | (11.40) | 273. | (13.14) | 1.18 | (0.98 - 1.41) | 0.079 |
| Parental separation (yes) | 0% | 1271. | (54.27) | 1380. | (47.47) | 0.76 | (0.68 - 0.85) | <0.0001 |
| Parental smoking (yes) | 16% | 1687. | (72.03) | 1532. | (73.55) | 1.08 | (0.95 - 1.23) | 0.259 |
| Parental alcohol use (yes) | 16% | 1618. | (69.09) | 1364. | (65.48) | 0.85 | (0.75 - 0.96) | 0.011 |
| Father-child relationship (1 (worst) - 5) | 18% | 2342 | 4.15 (1.16) | 1937 | 4.03 (1.22) | 0.92 | (0.87 - 0.97) | 0.001 |
| Mother-child relationship (1 (worst) - 5) | 17% | 2342 | 4.49 (0.85) | 1997 | 4.35 (0.97) | 0.84 | (0.79 - 0.90) | <0.0001 |
| Hyperactivity problems (0 - 10) | 16% | 2342 | 4.10 (3.09) | 2066 | 4.58 (3.08) | 1.05 | (1.03 - 1.07) | <0.0001 |

N = number/frequency, OR = Odds Ratio, 95%CI = 95% Confidence Interval, SD = Standard deviation, complete = no missing information

# **Appendix S3.** Details on multiple imputation

To address missing data, we used multivariate imputation by chained equations^33^ to impute all incomplete analysis variables up to our starting sample (those that provided data on alcohol use and drug use from at least one time point between age 11 and age 22 years; N = 4,599). Despite the univariable association observed between conduct problems and missingness (suggesting that the exposure could be missing-not-at-random), we decided to impute the small amount of missing data on conduct problems within our starting sample (<5% missing) given the strong auxiliary data and wide range of confounders available for our imputation model, making the missing-at-random assumption plausible. First, we investigated the patterns of missing data, the percentage of missingness for each analysis variable and potential auxiliary variables and determined the distribution of missing variables (see Supplementary material (Table S2)). Next, we performed multivariable regression analyses with each incomplete analysis variable as the outcome and potential auxiliary variables (such as earlier assessments of the mediators and outcomes) as the exposures to determine which auxiliary variables should be used to impute which analysis variables. In the imputation model, all analysis variables (exposure, mediators, outcomes, and confounders) were included in the imputation equation for every imputed variable. Additionally, we used tailored imputation equations to include only the auxiliary variables which were associated with the variable to be imputed in the regression models described above.

We used the Stata command -mi impute chained- with 10 cycles of regression switching and generated 40 imputed datasets. We imputed binary variables using binary logistic regression models, ordinal variables using ordinal logistic regression models, and all continuous variables using predicitve mean matching (with 10 nearest-neighbours) given evidence for a skewed distribution (Supplementary material (Table S2)). Given that gang membership was a composite of two time points, gang membership at age 18 and at age 22 were imputed seperately and then combined to passively impute the composite measure of gang membership at either age, which was then included in the imputation equations for all analysis variables. After data were imputed, we compared the summary statistics (mean or proportion) for each analysis variable across complete case and imputed data to identify if there were any large differences. Convergence plots were also used to ensure that 10 cycles of regression switching was sufficient, and Monte Carlo errors were examined in the regression models for the outcomes and mediators to ensure that 40 imputed datasets were sufficient^33^.

After imputation, all analyses were combined across the 40 datasets using Rubin’s rules. For descriptive statistics and regression models, this was done using the Stata command -mi estimate-. Mediation models were estimated across each imputed dataset using a loop, with the results from each imputed dataset saved into a logfile. The log odds ratios and bootstrapped standard errors for the mediation effects (total causal effect, natural indirect effect, and natural direct effect) across each imputed dataset were extracted from the logfile. The mean of the log odds ratio for each mediation effect across the 40 imputed datasets was calculated, and the standard error was calculated using Rubin’s rules. Imputation and subsequent analysis code for the mediation models can be found on GitHub: <https://github.com/gemmahammerton/gformula_1993_Pelotas>

# **Table S2.** Summary of missing data for analysis and auxiliary variables

| Variable | Description | N(%) missing (within total sample^1^) | N(%) missing (within imputed^2^) | Range | Imputation model^3^ | Skew | Mean/ % before imputation^4^ | Mean/ % after imputation^5^ |
| --- | --- | --- | --- | --- | --- | --- | --- | --- |
| **Main Analysis Variables** | | | | | | |  |  |
| conduct_11 | High (4+) conduct problems (SDQ) at age 11 years | 844 (16·1%) | 226 (4·9%) | 0/1 | logit | - | 31·1% | 30·8% |
| audit_haz_22 | Hazardous alcohol use at age 22 years | 1447 (27·6%) | 846 (18·4%) | 0/1 | logit | - | 21·6% | 22·3% |
| drugs_22 | Current illegal drug use at age 22 years | 1747 (33·3%) | 1097 (23·9%) | 0/1 | logit | - | 13·5% | 14·1% |
| arrest_18 | Ever been arrested or detained by age 18 years | 1145 (21·8%) | 523 (11·4%) | 0/1 | logit | - | 3·5% | 3·6% |
| gang_18_22 | Gang membership in last year at age 18 or 22 | 1229 (23·4%) | 594 (12·9%) | 0/1 | logit | - | 1·9% | 2·3% |
| edu_22 | Not completed school by age 22 years | 1444 (27·5%) | 843 (18·3%) | 0/1 | logit | - | 41·5% | 43·2% |
| cigf_15 | Frequency of cigarette smoking at age 15 years | 1025 (19·5%) | 384 (8·4%) | 0-2 | ologit | - | 0.24 | 0.24 |
| alcf_15 | Frequency of alcohol use at age 15 years | 1058 (20·2%) | 416 (9·1%) | 0-2 | ologit | - | 0.82 | 0.82 |
| peer_drugs_15 | Peers taken an illegal drug at age 15 years | 1498 (28·5%) | 852 (18·5%) | 0/1 | logit | - | 12·5% | 12·8% |
| peer_dev_11 | Friends are a bad influence at age 11 years | 827 (15·8%) | 213 (4·6%) | 0/1 | logit | - | 46·6% | 46·6% |
| female | Female sex at birth | 1 (0.02%) | 0 (0%) | 0/1 | n/a | - | 50·7% | 50·7% |
| rs_soc | Social risk score at birth | 0 (0%) | 0 (0%) | 0-5 | n/a | <0·001 | 0.90 | 0.90 |
| rs_bio | Health risk score at birth | 577 (11·0%) | 476 (10·4%) | 0-6 | pmm | <0·001 | 1.54 | 1.56 |
| matdep_11 | Maternal depression symptoms at age 11 years | 847 (16·1%) | 234 (5·1%) | 0-20 | pmm | <0·001 | 5.72 | 5.69 |
| fear_11 | Fear of the neighbourhood at age 11 years | 829 (15·8%) | 216 (4·7%) | 0/1 | logit | - | 12·2% | 12·2% |
| sep_11 | Parental separation at age 11 years | 0 (0%) | 0 (0%) | 0/1 | n/a | - | 57·2% | 57·2% |
| par_smoke_11 | Either parent smoked cigarettes by child age 11 | 824 (15·7%) | 211 (4·6%) | 0/1 | logit | - | 72·7% | 72·8% |
| par_alc_11 | Either parent drinks alcohol at child age 11 | 824 (15·7%) | 211 (4·6%) | 0/1 | logit | - | 67·5% | 67·7% |
| mum_rel_11 | Quality of relationship with mum at age 11 years | 910 (17·3%) | 272 (5·9%) | 1-5 | pmm | <0·001 | 4.43 | 4.43 |
| dad_rel_11 | Quality of relationship with dad at age 11 years | 970 (18·5%) | 330 (7·2%) | 1-5 | pmm | <0·001 | 4.10 | 4.10 |
| **Secondary Analysis Variables** | | | | | | |  |  |
| hyper_11 | Hyperactivity problems (SDQ) at age 11 years | 841 (16·0%) | 223 (4·9%) | 0-10 | pmm | <0·001 | 4.30 | 4.30 |
| emo_15 | Emotional problems (SDQ) at age 15 years | 913 (17·4%) | 307 (6·7%) | 0-10 | pmm | <0·001 | 3.93 | 3.93 |
| **Auxiliary Variables** | | | | | | |  |  |
| a_malcohol | Maternal alcohol consumption in pregnancy | 0 (0%) | 0 (0%) | 0/1 | n/a | - | 5·1% | 5·1% |
| a_msmoke | Maternal smoking in pregnancy | 0 (0%) | 0 (0%) | 0/1 | n/a | - | 33·3% | 33·3% |
| alc_11 | Frequency of alcohol use at age 11 years | 869 (16·7%) | 231 (5·0%) | 0-3 | ologit | - | 0.23 | 0.23 |
| cig_11 | Frequency of cigarette smoking at age 11 years | 877 (16·7%) | 239 (5·2%) | 0-3 | ologit | - | 0.04 | 0.05 |
| edu_11 | Last school grade completed at age 11 years | 807 (15·4%) | 188 (4·1%) | 0-7 | pmm | <0·001 | 3.63 | 3.53 |
| cpc_11 | Conduct problems (SDQ) at age 11 (child report) | 1213 (23·1%) | 569 (12·4%) | 0-10 | pmm | <0·001 | 2.26 | 2.30 |
| emo_11 | Emotional problems (SDQ) at age 11 years | 845 (16·1%) | 229 (5·0%) | 0-10 | pmm | <0·001 | 4.17 | 4.19 |
| fearc_11 | Fear of the neighbourhood at 11 (child report) | 816 (15·6%) | 191 (4·2%) | 0/1 | logit | - | 16·0% | 16·0% |
| edu_15 | Number of times repeated grade by age 15 years | 939 (17·9%) | 322 (7·0%) | 0-10 | pmm | <0·001 | 1.31 | 1.39 |
| cp_15 | Conduct problems (SDQ) at age 15 years | 911 (17·4%) | 306 (6·7%) | 0-10 | pmm | <0·001 | 2.23 | 2.28 |
| aces_15 | Adverse childhood experiences at age 15 | 1184 (22·6%) | 540 (11·7%) | 0-5 | pmm | <0·001 | 0.43 | 0.43 |
| drugs_15 | Any illegal drug use by age 15 | 1184 (22·6%) | 537 (11·7%) | 0/1 | logit | - | 1·6% | 1·7% |
| drugs_18 | Any illegal drug use by age 18 | 1980 (37·7%) | 1334 (29·0%) | 0/1 | logit | - | 26·3% | 28·3% |
| audit_18 | Alcohol-related problems at age 18 years | 1893 (36·1%) | 1260 (27·4%) | 0-37 | pmm | <0·001 | 5.94 | 5.83 |
| edu_18 | Number of times repeated grade by age 18 years | 1247 (23·8%) | 625 (13·6%) | 0-14 | pmm | <0·001 | 1.81 | 1.83 |

^1^Full sample *N* = 5,249; ^2^Imputed sample *N* = 4,599; ^3^Regression model used to impute incomplete variables; logit=binary logistic regression, ologit=ordinal logistic regression; pmm=predictive mean matching; ^4^Using complete data on variable within sample to be imputed (*N* = 4,599); ^5^Using imputed data on variable within imputed sample (*N* = 4,599)

# **Table S3.** Univariable associations for baseline confounders with mediators and outcomes using imputed data; *N* = 4,599

| OR (95% CI); p value  **Baseline confounders** | Mediators | | | Outcomes | |
| --- | --- | --- | --- | --- | --- |
|  | Police arrest | Gang membership | School non-completion | Hazardous alcohol use | Illicit drug use |
| Female sex | 0.14 (0.09, 0.23); <0.001 | 0.19 (0.10, 0.35); <0.001 | 0.57 (0.50, 0.64); <0.001 | 0.38 (0.33, 0.45); <0.001 | 0.42 (0.34, 0.51); <0.001 |
| Sociodemographic risk score | 1.25 (1.06, 1.47); 0.007 | 1.42 (1.16, 1.75); 0.001 | 2.23 (2.07, 2.40); <0.001 | 0.93 (0.86, 1.01); 0.082 | 0.91 (0.83, 1.01); 0.069 |
| Health risk score | 1.19 (1.02, 1.40); 0.030 | 1.15 (0.93, 1.43); 0.189 | 1.50 (1.41, 1.60); <0.001 | 1.02 (0.95, 1.09); 0.620 | 1.00 (0.91, 1.09); 0.960 |
| Maternal depression | 1.06 (1.03, 1.10); 0.001 | 1.09 (1.05, 1.14); <0.001 | 1.10 (1.08, 1.12); <0.001 | 1.00 (0.98, 1.02); 0.807 | 1.00 (0.98, 1.03); 0.720 |
| Fear of the neighbourhood | 1.21 (0.75, 1.95); 0.424 | 1.37 (0.74, 2.56); 0.317 | 1.51 (1.25, 1.83); <0.001 | 0.81 (0.63, 1.04); 0.101 | 1.25 (0.96, 1.65); 0.101 |
| Parental separation | 2.39 (1.62, 3.53); <0.001 | 2.24 (1.37, 3.66); 0.001 | 2.95 (2.59, 3.36); <0.001 | 1.12 (0.95, 1.31); 0.174 | 1.17 (0.97, 1.42); 0.107 |
| Parental smoking | 1.61 (1.07, 2.44); 0.024 | 1.12 (0.68, 1.85); 0.666 | 1.70 (1.46, 1.97); <0.001 | 1.07 (0.89, 1.28); 0.455 | 1.03 (0.83, 1.28); 0.760 |
| Parental alcohol use | 1.02 (0.71, 1.46); 0.908 | 0.63 (0.40, 1.00); 0.051 | 0.87 (0.76, 1.00); 0.050 | 1.37 (1.16, 1.63); <0.001 | 1.32 (1.07, 1.64); 0.010 |
| Father-child relationship | 0.84 (0.74, 0.95); 0.005 | 0.82 (0.69, 0.97); 0.022 | 0.74 (0.70, 0.79); <0.001 | 0.96 (0.89, 1.02); 0.191 | 0.91 (0.84, 0.99); 0.024 |
| Mother-child relationship | 0.94 (0.78, 1.12); 0.469 | 0.65 (0.53, 0.78); <0.001 | 0.61 (0.57, 0.66); <0.001 | 0.91 (0.83, 0.99); 0.028 | 0.98 (0.87. 1.11); 0.764 |
| Hyperactivity problems | 1.15 (1.09, 1.21); <0.001 | 1.26 (1.17, 1.36); <0.001 | 1.20 (1.18, 1.23); <0.001 | 1.08 (1.06, 1.11); <0.001 | 1.04 (1.01, 1.07); 0.010 |

OR = Odds Ratio, 95% CI = 95% Confidence Interval

# **Table S4.** Univariable associations for intermediate confounders with mediators and outcomes using imputed data; *N* = 4,599

| OR (95% CI);  p value | Police arrest | Gang membership | School non-completion | Hazardous alcohol use | Illicit drug use |
| --- | --- | --- | --- | --- | --- |
| **Intermediate confounders** | | | | | |
| Peer deviance | 2.23 (1.56, 3.19); <0.001 | 2.91 (1.75, 4.86); <0.001 | 1.57 (1.38, 1.79); <0.001 | 1.39 (1.20, 1.62); <0.001 | 1.67 (1.36, 2.04); <0.001 |
| Frequency of cigarette smoking (never) | Reference | | | | |
| Not in last month | 2.22 (1.40, 3.53) | 1.46 (0.76, 2.81) | 1.69 (1.39, 2.04) | 1.07 (0.84, 1.36) | 1.81 (1.40, 2.36) |
| 1 to 5 days in last month | 3.81 (2.33, 6.22) | 2.45 (1.17, 5.11) | 3.29 (2.44, 4.45) | 1.78 (1.32, 2.40) | 1.89 (1.30, 2.75) |
| Frequency of alcohol use (never) | Reference | | | | |
| Not in last month | 1.78 (1.13, 2.80) | 1.27 (0.72, 2.26) | 0.69 (0.59, 0.80) | 1.45 (1.19, 1.75) | 2.20 (1.69, 2.86) |
| 1 to 5 days in last month | 2.30 (1.44, 3.67) | 1.61 (0.90, 2.87) | 0.84 (0.72, 0.99) | 2.00 (1.63, 2.45) | 3.12 (2.37, 4.09) |
| Peer drug use | 2.43 (1.57, 3.76); <0.001 | 2.47 (1.40, 4.38); 0.002 | 1.42 (1.14, 1.77); 0.002 | 1.39 (1.10, 1.76); 0.006 | 2.53 (1.97, 3.27); <0.001 |
| Emotional problems | 0.99 (0.93, 1.05); 0.700 | 1.02 (0.94, 1.10); 0.704 | 1.10 (1.07, 1.13); <0.001 | 0.97 (0.94, 1.00); 0.060 | 0.95 (0.92, 0.99); 0.010 |

OR = Odds Ratio, 95% CI = 95% Confidence Interval

# **Table S5.** Mediation models for school non-completion with police arrest and gang membership treated as intermediate confounders using imputed data; *N* = 4,599

| OR  (95% CI) | Adjusted for baseline and intermediate confounders^1^ | Additionally adjusted for comordidity^2^ |
| --- | --- | --- |
| **Hazardous alcohol use** | | |
| TCE | 1.20 (1.00, 1.44) | 1.03 (0.85, 1.26) |
| NDE | 1.19 (0.99, 1.43) | 1.03 (0.84, 1.26) |
| NIE | 1.01 (0.98, 1.03) | 1.01 (0.99, 1.02) |
| PM | 4% | n/a |
| **Illicit drug use** | | |
| TCE | 1.05 (0.83, 1.34) | 1.01 (0.80, 1.29) |
| NDE | 1.05 (0.82, 1.34) | 1.01 (0.79, 1.28) |
| NIE | 1.00 (0.96, 1.04) | 1.01 (0.98, 1.03) |
| PM | n/a | n/a |

OR = Odds Ratio, 95% CI = 95% Confidence Interval, TCE = Total Causal Effect, NDE = Natural Direct Effect, NIE= Natural Indirect Effect, PM = Proportion mediated, n/a = not calculated due to inconsistent mediation or a very small total effect; ^1^ adjusted for baseline confounders including sex, score of sociodemographic risk factors, score of health risk factors (all measured perinatally), maternal depression, father/child relationship, mother/child relationship, parental alcohol consumption, parental smoking, parental separation, neighbourhood safety (all measured at age 11) and intermediate confounders including peer deviance (at age 11), frequency of cigarette smoking (at age 15), frequency of alcohol drinking (at age 15), peer drug use (at age 15), police arrest (age 18), gang membership (age 18 and 22); ^2^ adjusted for baseline and intermediate confounders listed above, and additionally adjusted for hyperactivity problems at age 11 as a baseline confounder and emotional problems at age 15 as an intermediate confounder.

# **Table S6.** Associations between the exposure (conduct problems at age 11 years) and the mediators (police arrest, gang membership, and school non-completion between age 18 and 22 years) using complete case data; N = 2*,*342

| OR (95% CI);  p value | Unadjusted | Adjusted for baseline confounders^1^ | Additionally adjusted for hyperactivity^2^ |
| --- | --- | --- | --- |
| Police arrest | 1.91 (1.14, 3.17); 0.013 | 1.47 (0.85, 2.53); 0.166 | 1.16 (0.65, 2.08); 0.617 |
| Gang membership | 2.55 (1.31, 4.98); 0.006 | 1.78 (0.87, 3.64); 0.112 | 1.25 (0.58, 2.70); 0.561 |
| School non-completion | 2.64 (2.19, 3.18); <0.001 | 1.89 (1.53, 2.34); <0.001 | 1.54 (1.23, 1.94); <0.001 |

OR = Odds Ratio, 95% CI = 95% Confidence Interval; ^1^ adjusted for baseline confounders including sex, score of sociodemographic risk factors, score of health risk factors (all measured perinatally), maternal depression, father/child relationship, mother/child relationship, parental alcohol consumption, parental smoking, parental separation, neighbourhood safety (all measured at age 11); ^2^ adjusted for baseline confounders listed above, and additionally adjusted for hyperactivity problems at age 11

# **Table S7.** Associations between the mediators (police arrest, gang membership, and school non-completion between age 18 and 22 years) and the outcomes (hazardous alcohol use and illicit drug use at age 22 years) using complete case data; N = 2*,*342

| OR (95% CI);  p value | Unadjusted | Adjusted for baseline and intermediate confounders^1^ | Additionally adjusted for comordidity^2^ | Additionally adjusted for all mediators |
| --- | --- | --- | --- | --- |
| **Hazardous alcohol use** | | | | |
| Police arrest | 3.22 (1.94, 5.36); <0.001 | 2.10 (1.22, 3.60); 0.007 | 2.02 (1.17, 3.48); 0.011 | 1.79 (1.02, 3.13); 0.041 |
| Gang membership | 4.22 (2.16, 8.25); <0.001 | 2.82 (1.38, 5.77); 0.004 | 2.70 (1.32, 5.53); 0.007 | 2.35 (1.12, 4.91); 0.023 |
| School non-completion | 1.25 (1.02, 1.53); 0.035 | 1.17 (0.92, 1.49); 0.206 | 1.13 (0.88, 1.44); 0.332 | 1.08 (0.85, 1.39); 0.522 |
| **Illicit drug use** | | | | |
| Police arrest | 6.62 (3.98, 11.03); <0.001 | 4.44 (2.56, 7.73); <0.001 | 4.38 (2.52, 7.63); <0.001 | 3.64 (2.03, 6.52); <0.001 |
| Gang membership | 12.10 (6.03, 24.30); <0.001 | 9.00 (4.27, 18.96); <0.001 | 8.86 (4.20, 18.70); <0.001 | 7.33 (3.37, 15.92); <0.001 |
| School non-completion | 1.24 (0.97, 1.59); 0.084 | 1.14 (0.85, 1.52); 0.378 | 1.12 (0.84, 1.50); 0.436 | 1.00 (0.74, 1.35); 0.982 |

OR = Odds Ratio, 95% CI = 95% Confidence Interval; ^1^ adjusted for high conduct problems at age 11 and baseline confounders including sex, score of sociodemographic risk factors, score of health risk factors (all measured perinatally), maternal depression, father/child relationship, mother/child relationship, parental alcohol consumption, parental smoking, parental separation, neighbourhood safety (all measured at age 11) and intermediate confounders including peer deviance (at age 11), frequency of cigarette smoking (at age 15), frequency of alcohol drinking (at age 15), peer drug use (at age 15); ^2^ adjusted for baseline and intermediate confounders listed above, and additionally adjusted for hyperactivity problems at age 11 as a baseline confounder and emotional problems at age 15 as an intermediate confounder.

# **Table S8.** Mediation models including all mediators (police arrest, gang membership, and school non-completion) simultaneously using complete case data; N = 2*,*342

| OR  (95% CI) | Unadjusted | Adjusted for baseline and intermediate confounders^1^ | Additionally adjusted for comordidity^2^ | |
| --- | --- | --- | --- | --- |
| **Hazardous alcohol use** | | | |  |
| TCE | 1.29 (1.04, 1.61) | 1.23 (1.00, 1.53) | 1.08 (0.82, 1.42) | |
| NDE | 1.22 (0.97, 1.52) | 1.19 (0.95, 1.49) | 1.06 (0.80, 1.40) | |
| NIE | 1.06 (1.00, 1.13) | 1.04 (0.99, 1.08) | 1.02 (0.99, 1.05) | |
| PM | 24% | 17% | 22% | |
| **Illicit drug use** | | | |  |
| TCE | 1.18 (0.95, 1.48) | 1.07 (0.82, 1.40) | 0.99 (0.73, 1.35) | |
| NDE | 1.08 (0.85, 1.36) | 1.01 (0.76, 1.34) | 0.97 (0.71, 1.32) | |
| NIE | 1.10 (0.99, 1.22) | 1.06 (0.99, 1.14) | 1.02 (0.96, 1.10) | |
| PM | 57% | 87% | n/a | |

OR = Odds Ratio, 95% CI = 95% Confidence Interval, TCE = Total Causal Effect, NDE = Natural Direct Effect, NIE= Natural Indirect Effect, PM = Proportion mediated, n/a = not calculated due to inconsistent mediation; ^1^ adjusted for baseline confounders including sex, score of sociodemographic risk factors, score of health risk factors (all measured perinatally), maternal depression, mother/child relationship, father/child relationship, parental alcohol consumption, parental smoking, parental separation, neighbourhood safety (all measured at age 11) and intermediate confounders including peer deviance (at age 11), frequency of cigarette smoking (at age 15), frequency of alcohol drinking (at age 15), peer drug use (at age 15); ^32^adjusted for baseline and intermediate confounders listed above, and additionally adjusted for hyperactivity problems at age 11 as a baseline confounder and emotional problems at age 15 as an intermediate confounder.

# **Table S9.** Associations between the mediators (police arrest, gang membership, and school non-completion between age 18 and 22 years) and the AUDIT subscales (alcohol consumption and alcohol problems at age 22 years) using imputed data; *N* = 4,599

| beta (95% CI);  p value | Unadjusted | Adjusted for baseline and intermediate confounders^1^ | Additionally adjusted for comordidity^2^ | Additionally adjusted for all mediators |
| --- | --- | --- | --- | --- |
| **Alcohol consumption** | | | | |
| Police arrest | 1.57 (1.06, 2.07); <0.001 | 0.88 (0.38, 1.38); 0.001 | 0.86 (0.36, 1.36); 0.001 | 0.77 (0.27, 1.27); 0.003 |
| Gang membership | 1.35 (0.65, 2.06); <0.001 | 0.83 (0.14, 1.53); 0.019 | 0.79 (0.10, 1.49); 0.025 | 0.63 (-0.06, 1.32); 0.072 |
| School non-completion | 0.21 (0.04, 0.38); 0.018 | 0.14 (-0.06, 0.33); 0.163 | 0.11 (-0.08, 0.30); 0.264 | 0.08 (-0.12, 0.27); 0.439 |
| **Alcohol problems** | | | | |
| Police arrest | 1.32 (0.69, 1.95); <0.001 | 0.70 (0.07, 1.32); 0.030 | 0.69 (0.06, 1.32); 0.032 | 0.59 (-0.05, 1.22); 0.069 |
| Gang membership | 1.38 (0.62, 2.14); <0.001 | 0.84 (0.08, 1.61); 0.031 | 0.82 (0.05, 1.58); 0.037 | 0.69 (-0.09, 1.46); 0.081 |
| School non-completion | 0.34 (0.14, 0.55); 0.001 | 0.15 (-0.08, 0.38); 0.204 | 0.11 (-0.12, 0.35); 0.328 | 0.09 (-0.15, 0.32); 0.472 |

beta = unstandardised beta coefficient, 95% CI = 95% Confidence Interval; ^1^ adjusted for high conduct problems at age 11 and baseline confounders including sex, score of sociodemographic risk factors, score of health risk factors (all measured perinatally), maternal depression, father/child relationship, mother/child relationship, parental alcohol consumption, parental smoking, parental separation, neighbourhood safety (all measured at age 11) and intermediate confounders including peer deviance (at age 11), frequency of cigarette smoking (at age 15), frequency of alcohol drinking (at age 15), peer drug use (at age 15); ^2^ adjusted for baseline and intermediate confounders listed above, and additionally adjusted for hyperactivity problems at age 11 as a baseline confounder and emotional problems at age 15 as an intermediate confounder.

# **Table S10.** Mediation models including all mediators (police arrest, gang membership, and school non-completion) simultaneously using imputed data; *N* = 4,599

| b  (95% CI) | Unadjusted | Adjusted for baseline and intermediate confounders^1^ | Additionally adjusted for comordidity^2^ |  |
| --- | --- | --- | --- | --- |
| **Alcohol consumption** | | | | |
| TCE | 0.28 (0.08, 0.47) | 0.20 (0.01, 0.39) | 0.09 (-0.14, 0.32) |  |
| NDE | 0.20 (-0.01, 0.40) | 0.17 (-0.02, 0.36) | 0.08 (-0.15, 0.30) |  |
| NIE | 0.08 (0.03, 0.14) | 0.03 (0.001, 0.06) | 0.01 (-0.01, 0.04) |  |
| PM | 29% | 16% | 15% |  |
| **Alcohol problems** | | | | |
| TCE | 0.36 (0.15, 0.57) | 0.22 (-0.01, 0.44) | 0.10 (-0.16, 0.36) |  |
| NDE | 0.25 (0.03, 0.48) | 0.18 (-0.04, 0.40) | 0.09 (-0.17, 0.34) |  |
| NIE | 0.11 (0.05, 0.17) | 0.03 (-0.004, 0.07) | 0.01 (-0.01, 0.04) |  |
| PM | 30% | 15% | 13% |  |

b = unstandardised beta coefficient, 95% CI = 95% Confidence Interval, TCE = Total Causal Effect, NDE = Natural Direct Effect, NIE= Natural Indirect Effect, PM = Proportion mediated, n/a = not calculated due to inconsistent mediation or a very small total effect; ^1^ adjusted for baseline confounders including sex, score of sociodemographic risk factors, score of health risk factors (all measured perinatally), maternal depression, father/child relationship, mother/child relationship, parental alcohol consumption, parental smoking, parental separation, neighbourhood safety (all measured at age 11) and intermediate confounders including peer deviance (at age 11), frequency of cigarette smoking (at age 15), frequency of alcohol drinking (at age 15), peer drug use (at age 15); ^2^ adjusted for baseline and intermediate confounders listed above, and additionally adjusted for hyperactivity problems at age 11 as a baseline confounder and emotional problems at age 15 as an intermediate confounder.

# **Supplementary references**

1. Murray J, Irving B, Farrington DP, Colman I, Bloxsom CAJ. Very early predictors of conduct problems and crime: results from a national cohort study: Early predictors of conduct problems and crime. *Journal of Child Psychology and Psychiatry*. 2010;51(11):1198-1207. doi:10.1111/j.1469-7610.2010.02287.x

2. Murray J, Maughan B, Menezes AMB, et al. Perinatal and sociodemographic factors at birth predicting conduct problems and violence to age 18 years: comparison of Brazilian and British birth cohorts. *Child Psychology Psychiatry*. 2015;56(8):914-922. doi:10.1111/jcpp.12369

3. Murray J, Shenderovich Y, Gardner F, et al. Risk Factors for Antisocial Behavior in Low- and Middle-Income Countries: A Systematic Review of Longitudinal Studies. *Crime and Justice*. 2018;47(1):255-364. doi:10.1086/696590

4. Martins-Silva T, Bauer A, Matijasevich A, et al. Early risk factors for conduct problem trajectories from childhood to adolescence: the 2004 Pelotas (BRAZIL) Birth Cohort. *Eur Child Adolesc Psychiatry*. Published online April 25, 2023. doi:10.1007/s00787-023-02178-9

5. Howell JC, Egley A. Moving Risk Factors into Developmental Theories of Gang Membership. *Youth Violence and Juvenile Justice*. 2005;3(4):334-354. doi:10.1177/1541204005278679

6. Peterson D, Morgan KA. Sex differences and the overlap in youths’ risk factors for onset of violence and gang involvement. *Journal of Crime and Justice*. 2014;37(1):129-154. doi:10.1080/0735648X.2013.830393

7. Higginson A, Benier K, Shenderovich Y, Bedford L, Mazerolle L, Murray J. Factors associated with youth gang membership in low‐ and middle‐income countries: a systematic review. *Campbell Systematic Reviews*. 2018;14(1):1-128. doi:10.4073/csr.2018.11

8. Gubbels J, Van Der Put CE, Assink M. Risk Factors for School Absenteeism and Dropout: A Meta-Analytic Review. *J Youth Adolescence*. 2019;48(9):1637-1667. doi:10.1007/s10964-019-01072-5

9. Griffin KW, Botvin GJ. Evidence-Based Interventions for Preventing Substance Use Disorders in Adolescents. *Child and Adolescent Psychiatric Clinics of North America*. 2010;19(3):505-526. doi:10.1016/j.chc.2010.03.005

10. Mahedy L, MacArthur GJ, Hammerton G, et al. The effect of parental drinking on alcohol use in young adults: the mediating role of parental monitoring and peer deviance. *Addiction*. 2018;113(11):2041-2050. doi:10.1111/add.14280

11. Locatelli D, Sanchez Z, Opaleye E, Carlini C, Noto A. Socioeconomic influences on alcohol use patterns among private school students in São Paulo. *Revista Brasileira de Psiquiatria*. 2012;34(2):193-200. doi:10.1590/S1516-44462012000200012

12. Fergusson DM, Boden JM, Horwood LJ. The developmental antecedents of illicit drug use: Evidence from a 25-year longitudinal study. *Drug and Alcohol Dependence*. 2008;96(1-2):165-177. doi:10.1016/j.drugalcdep.2008.03.003

13. Tavares BF, Béria JU, Silva de Lima M. Factors associated with drug use among adolescent students in southern Brazil. *Rev Saúde Pública*. 2004;38(6):787-796. doi: 10.1590/s0034-89102004000600006.

14. Kramer MS, Platt RW, Wen SW, et al. A New and Improved Population-Based Canadian Reference for Birth Weight for Gestational Age. *Pediatrics*. 2001;108(2):e35-e35. doi:10.1542/peds.108.2.e35

15. De Jesus Mari J, Williams P. A validity study of a psychiatric screening questionnaire (SRQ-20) in primary care in the city of Sao Paulo. *British Journal of Psychiatry*. 1986;148(JAN.):23-26. doi:10.1192/bjp.148.1.23

16. Hingson RW, Heeren T, Winter MR. Age at drinking onset and alcohol dependence: Age at onset, duration, and severity. *Archives of Pediatrics and Adolescent Medicine*. 2006;160(7):739-746. doi:10.1001/archpedi.160.7.739

17. Pitkänen T, Lyyra AL, Pulkkinen L. Age of onset of drinking and the use of alcohol in adulthood: A follow-up study from age 8-42 for females and males. *Addiction*. 2005;100(5):652-661. doi:10.1111/j.1360-0443.2005.01053.x

18. Moffitt TE. Adolescence-Limited and Life-Course-Persistent Antisocial Behavior: A Developmental Taxonomy. *Psychological Review*. 1993;100(4).

19. Heron J, Maughan B, Dick DM, et al. Conduct problem trajectories and alcohol use and misuse in mid to late adolescence. *Drug and Alcohol Dependence*. 2013;133(1):100-107. doi:10.1016/j.drugalcdep.2013.05.025

20. Erskine HE, Norman RE, Ferrari AJ, et al. Long-Term Outcomes of Attention-Deficit/Hyperactivity Disorder and Conduct Disorder: A Systematic Review and Meta-Analysis. *Journal of the American Academy of Child & Adolescent Psychiatry*. 2016;55(10):841-850. doi:10.1016/j.jaac.2016.06.016

21. Kulkarni T, Sullivan AL, Kim J. Externalizing Behavior Problems and Low Academic Achievement: Does a Causal Relation Exist? *Educ Psychol Rev*. 2021;33(3):915-936. doi:10.1007/s10648-020-09582-6

22. Luderer M, Ramos Quiroga JA, Faraone SV, Zhang-James Y, Reif A. Alcohol use disorders and ADHD. *Neuroscience & Biobehavioral Reviews*. 2021;128:648-660. doi:10.1016/j.neubiorev.2021.07.010

23. Fergusson DM, Horwood LJ, Ridder EM. Conduct and attentional problems in childhood and adolescence and later substance use, abuse and dependence: Results of a 25-year longitudinal study. *Drug and Alcohol Dependence*. 2007;88:S14-S26. doi:10.1016/j.drugalcdep.2006.12.011

24. Treur JL, Munafò MR, Logtenberg E, Wiers RW, Verweij KJH. Using Mendelian randomization analysis to better understand the relationship between mental health and substance use: a systematic review. *Psychol Med*. 2021;51(10):1593-1624. doi:10.1017/S003329172100180X

25. Goodman R. The Strengths and Difficulties Questionnaire: A Research Note. *Journal of Child Psychology and Psychiatry*. 1997;38(5):581-586. doi:10.1111/j.1469-7610.1997.tb01545.x

26. Clayborne Z, Varin M, Colman I. Systematic Review and Meta-Analysis: Adolescent Depression and Long-Term Psychosocial Outcomes. *Journal of the American Academy of Child & Adolescent Psychiatry*. 2019;58(1):72-79.

27. Hammerton G, Murray J, Maughan B, et al. Childhood Behavioural Problems and Adverse Outcomes in Early Adulthood: a Comparison of Brazilian and British Birth Cohorts. *J Dev Life Course Criminology*. 2019;5(4):517-535. doi:10.1007/s40865-019-00126-3

28. Hoffmann MS, McDaid D, Salum GA, et al. The impact of child psychiatric conditions on future educational outcomes among a community cohort in Brazil. *Epidemiol Psychiatr Sci*. 2021;30:e69. doi:10.1017/S2045796021000561

29. Dyer ML, Easey KE, Heron J, Hickman M, Munafò MR. Associations of child and adolescent anxiety with later alcohol use and disorders: a systematic review and meta‐analysis of prospective cohort studies. *Addiction*. 2019;114(6):968-982. doi:10.1111/add.14575

30. Bevilacqua L, Hale D, Barker ED, Viner R. Conduct problems trajectories and psychosocial outcomes: a systematic review and meta-analysis. *Eur Child Adolesc Psychiatry*. 2018;27(10):1239-1260. doi:10.1007/s00787-017-1053-4

31. Schuster NA, Twisk JWR, Ter Riet G, Heymans MW, Rijnhart JJM. Noncollapsibility and its role in quantifying confounding bias in logistic regression. *BMC Med Res Methodol*. 2021;21(1):136. doi:10.1186/s12874-021-01316-8

32. VanderWeele TJ. *Explanation in Causal Inference: Methods for Mediation and Interaction*. Oxford University Press; 2015.

33. White IR, Royston P, Wood AM. Multiple imputation using chained equations: Issues and guidance for practice. *Statist Med*. 2011;30(4):377-399. doi:10.1002/sim.4067
